# FACILITY AND RESIDENT LEVEL DIFFERENCES: ANTIBIOTIC USE IN NURSING HOMES FOR RESIDENTS WITH ADVANCE DEMENTIA

**DOI:** 10.1093/geroni/igz038.3233

**Published:** 2019-11-08

**Authors:** Meghan Hendricksen, Daniel Habtemariam, Susan Mitchell

**Affiliations:** 1 Hebrew SeniorLife, Marcus Institute for Aging Research, Boston, Massachusetts, United States; 2 Hebrew SeniorLife, Institute for Aging Research, Boston, Massachusetts, United States

## Abstract

Previous studies have shown that there is a high frequency of antibiotic use in NH for advance dementia patients. However, research has shown limited clinical benefit from antimicrobial use for this population, and antimicrobial exposure increases colonization with drug-resistant bacteria in nursing homes. The aim of this study was to identify NH and resident level characteristics associated with antibiotic use for patients with advance dementia. Using data from an ongoing cluster RCT in 28 Boston NHs; Trial to Reduce Antimicrobial use in Nursing home residents with Alzheimer’s disease and other Dementias (TRAIN-AD), testing a program intervention to improve management of infections in advanced dementia. These data are taken from baseline measurements 2 months prior to intervention, and individual nursing home residents with advance dementia are units of analysis (n = 425). We ran multivariable logistic regression model with antibiotic use as the outcome, adjusting for clustering at NH level, with NH (#beds, profit status, staffing, #cognitively impaired, etc.) and individual patient characteristics (age, gender, race, etc.) as independent variables. Analyses found residents were more likely to receive antibiotics if they resided in nursing homes that employed less intense infectious disease practices prior to baseline (AOR = 2.34; 95% CI 1.08, 5.05), and full-time nurse practitioners or physician assistants (AOR= 3.68; 95%CI 1.49, 9.04). Female patients also had higher odds of receiving antibiotics (AOR=2.16; 95%CI1.10, 4.67). These findings provide potential insight into the importance of education regarding stringent infectious disease practices for practitioners, particularly for patients with advanced dementia.

